# Supplementary material for: Deep model predictive control of gene expression in thousands of single cells
Source: Nat Commun. 2024 Mar 8;15:2148. doi: 10.1038/s41467-024-46361-1 (PMC10923782; doi:10.1038/s41467-024-46361-1)
Supplement: Supplementary file 1 — Supplementary Information [file 41467_2024_46361_MOESM1_ESM.pdf]

SUPPLEMENTARY INFORMATION

Supplementary Figures

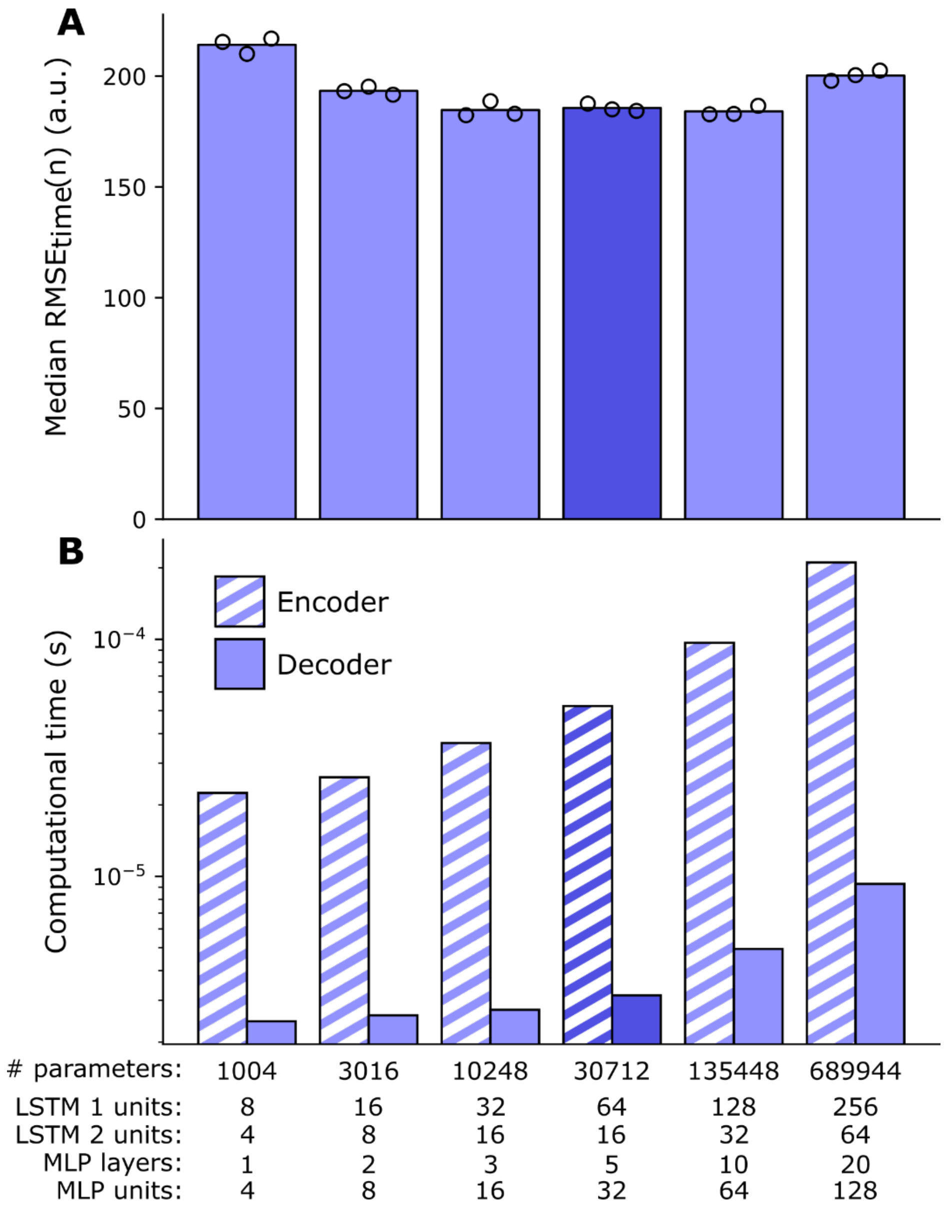

Figure S1. Evaluation of different sets of network hyperparameters. **(A)** Median $\mathrm{RMSE}_{\mathrm{time}}(n)$ for the different networks. Each network was trained from scratch and evaluated 3 times (black circles) and the blue bars represent the average of all 3 trainings. The darker bar represents the model parameters we used for the rest of this study. **(B)** Inference time for the different networks. The striped bar is for the encoder part of each network; solid bar is the decoder. Note that these computational times are different from experimental values reported in the Main Text because here all validation samples are evaluated at once and thus benefit from parallelization, while during experiments, data are processed for 27-28 cells at a time since that corresponds to the number of mother machine chambers in a single field of view.

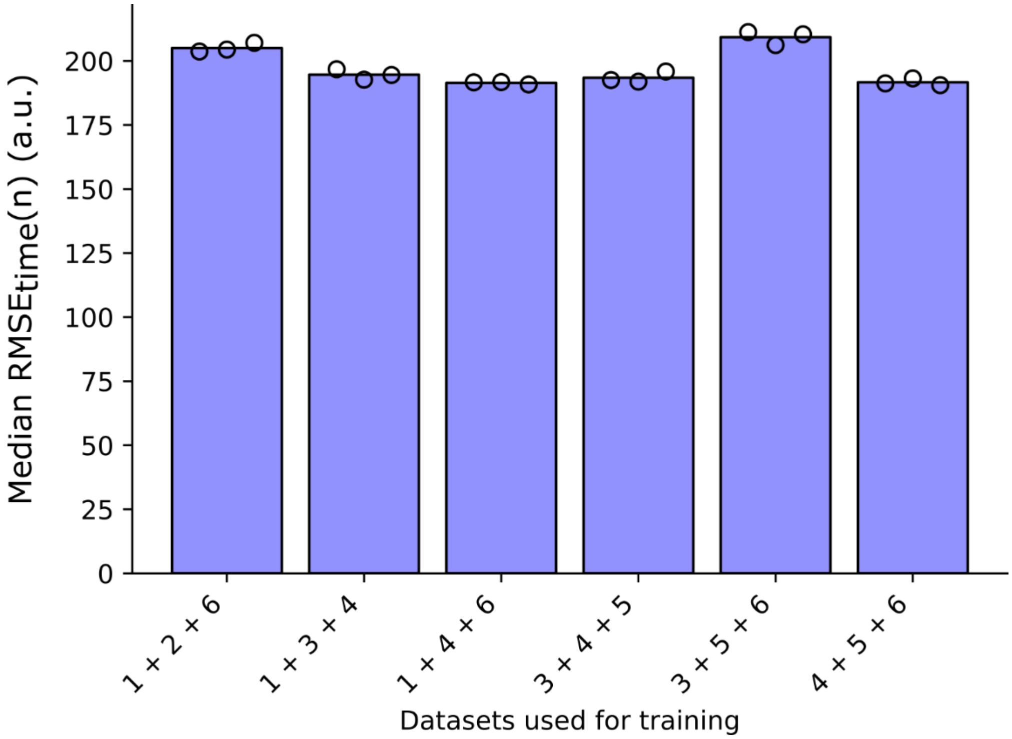

Figure S2. Median $\mathrm{RMSE}_{\mathrm{time}}(n)$ for different combinations of experimental datasets for training and validation. Dataset 7 was held out to compute the error shown here. To be able to shuffle the training data, we selected 3 experimental datasets out of the remaining 6 for training, as labelled on the x axis. For each set, the network was trained from scratch and evaluated 3 times (black circles) and the blue bars represent the average of all 3 trainings. In the rest of the study, we used datasets 1 – 4 for training and 5 – 7 for validation.

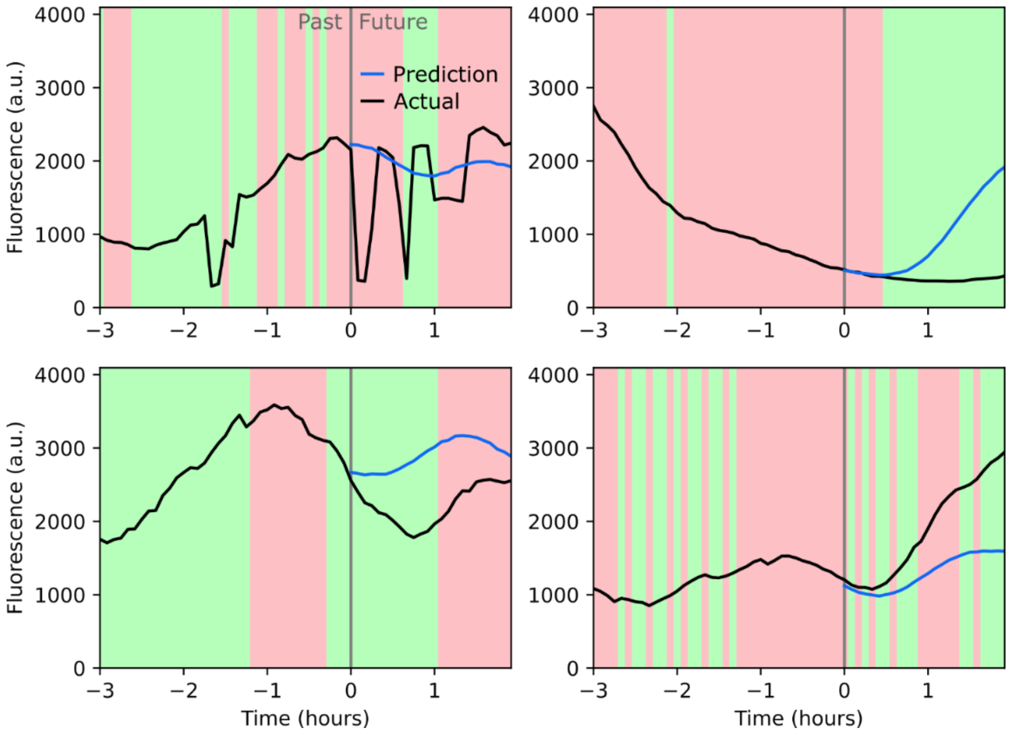

Figure S3. 95th percentile of $\mathrm{RMSE}_{\mathrm{time}}(n)$ for predictions for the 2-hour horizon model. Four representative examples are shown: In the top left panel, image analysis errors result in unpredictable jumps in fluorescence levels, and in the other 3 panels the cell "responsiveness" seems to suddenly change compared to past behavior. Red/green background colors represent optogenetic stimulations.

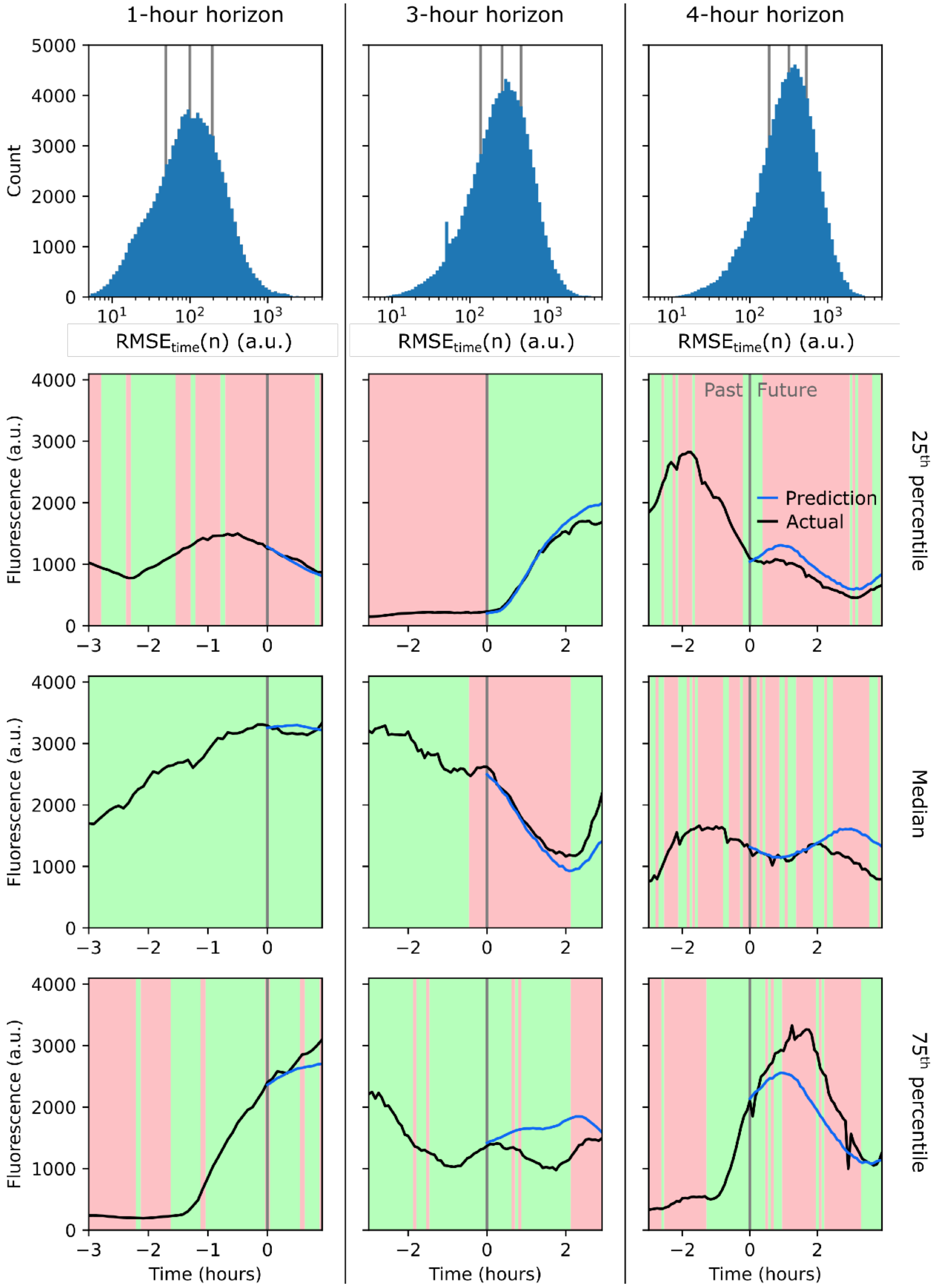

Figure S4. Performance of 1, 3, and 4-hour horizon models. Top row is the histogram of the $\mathrm{RMSE_{time}}(n)$ between 100,000 model predictions on the validation dataset and their ground truth. Vertical gray lines represent the 25th, median, and 75th percentile of the error, from top to bottom. Below each histogram are representative predictions at the 25th, median, and 75th percentile of the error for each of the different prediction horizon models.

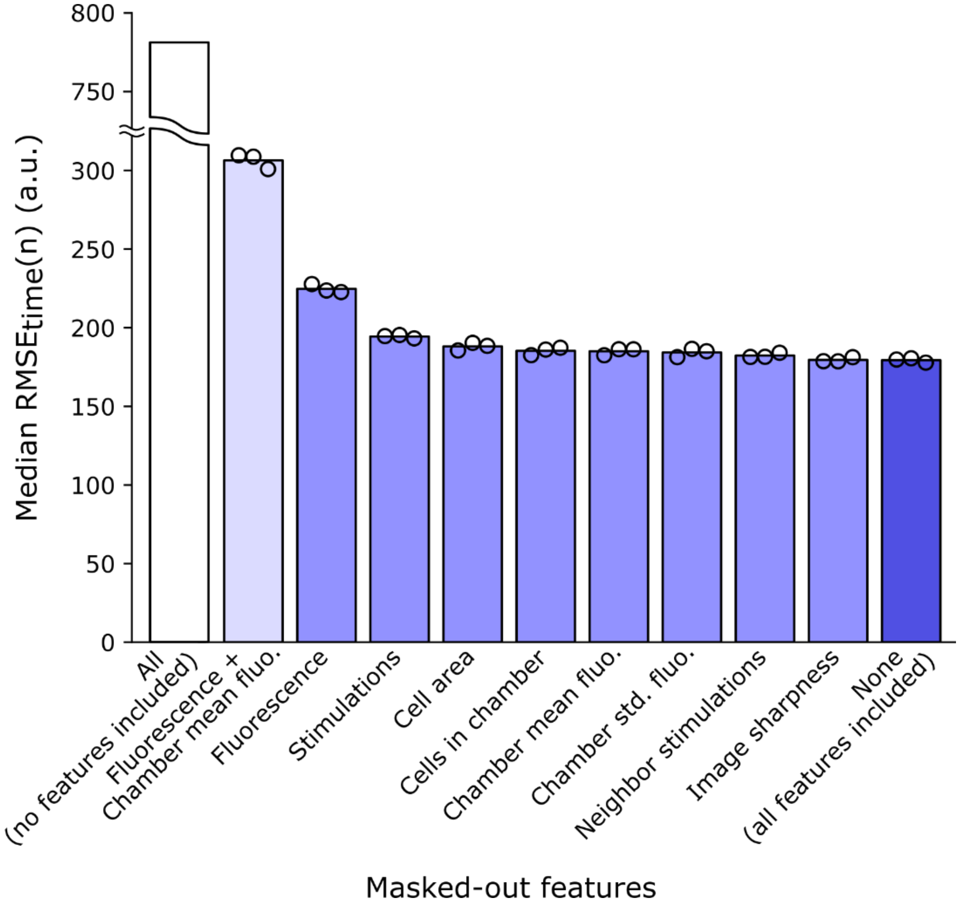

Figure S5. Median $\mathrm{RMSE}_{\mathrm{time}}(n)$ for masked-out features. The x axis lists which features were removed, except for the "None" case where all features are used. For every set of features, the network was trained from scratch and evaluated 3 times (black circles) and the blue bars represent the average of all 3 trainings. All networks were evaluated against the same test data, with masked-out input arrays. The darker bar represents the case we used for the rest of this study.

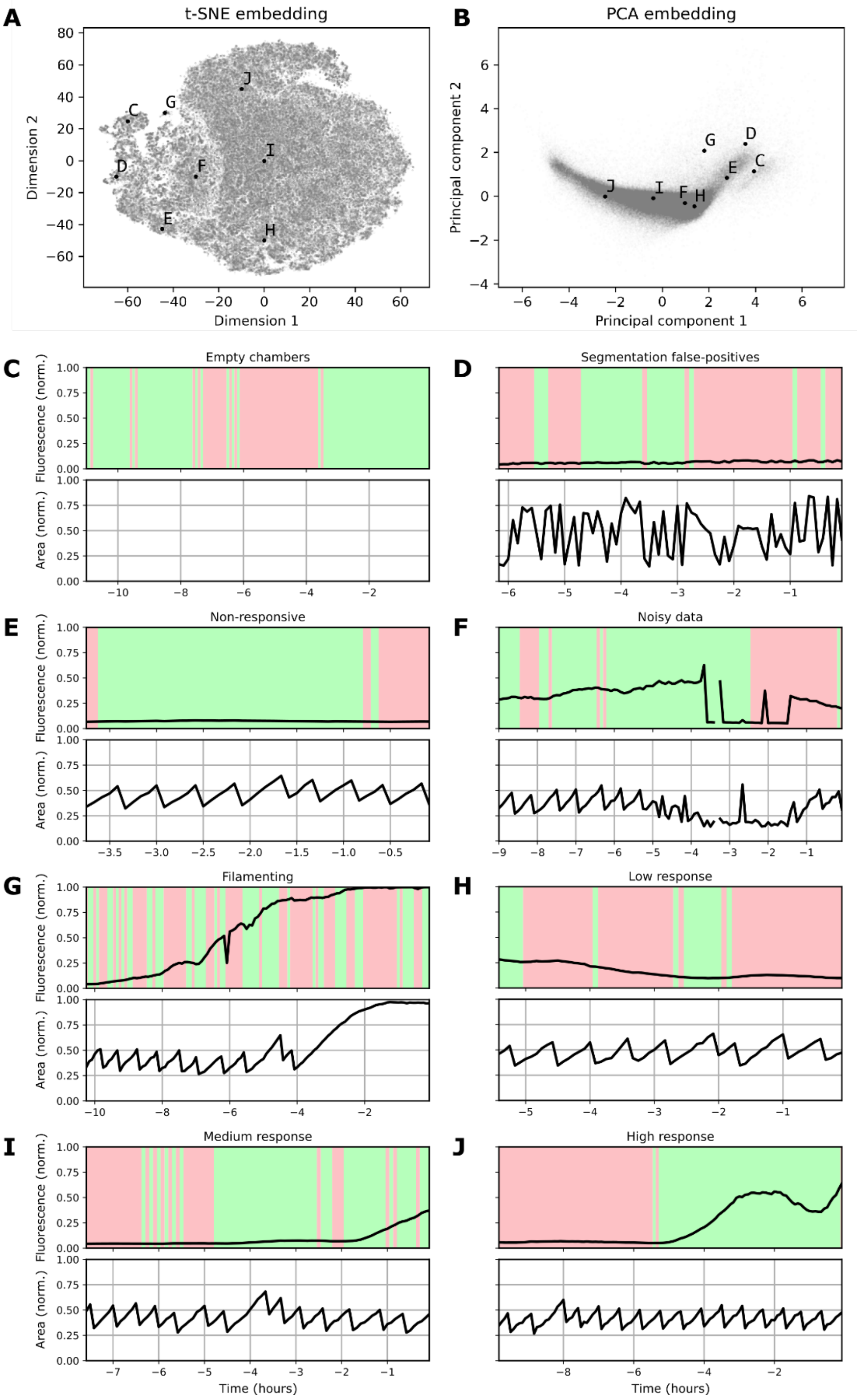

Figure S6. Embeddings of encoded latent space samples. **(A)** t-distributed stochastic neighbor embedding (t-SNE) of latent space encodings of cells' past. Cell data is not fundamentally categorical and does not result in clear clusters in the embedded space. However local trends can be identified, and the lettered

dots represent local illustrations of these trends shown in the other panels of this figure. **(B)** Principal Component Analysis (PCA) 2D embedding of the same data. The same samples are shown as lettered dots in (A) and (B). **(C-J)** Illustrations of local trends in the embeddings shown in panels (A) and (B). Title provides description of cell responses in this region. Note that trends tend to co-localize, for example errors such as empty chambers (C) and segmentation false positives (D) are separated from well-behaved outputs like cells with medium (I) or high (J) responses.

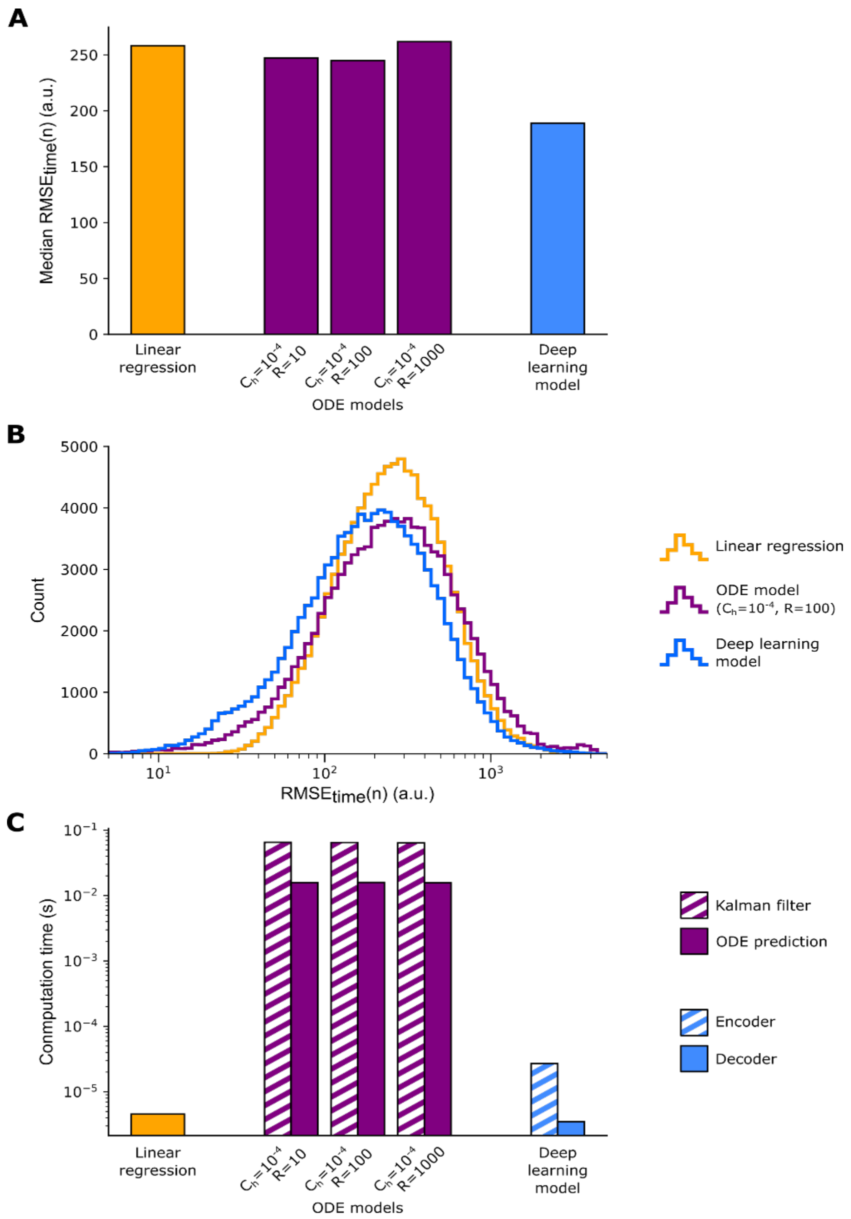

Figure S7. Evaluation of different timeseries forecasting models. **(A)** Median $\mathrm{RMSE}_{\mathrm{time}}(n)$ for the different models. Orange bar is the linear regression model, the three purple bars are the ODE-based model for the three combinations of technical parameters that minimize prediction error (Table S4, Supplementary Text). The blue bar is the model we used in the rest of this study. **(B)** $\mathrm{RMSE}_{\mathrm{time}}(n)$ distributions for the different models. For clarity only the best ODE model is shown. **(C)** Computation times for all the different models. For ODE models, the purple striped bars represent the time necessary to perform state estimation, while the solid purple bars represent the computation time for the future timeseries prediction that would need to be evaluated for every potential control strategy. For our LSTM-MLP deep learning model, the striped blue bar represents the computation time for the encoder part of the network, while the solid blue bar represents the time for the decoder part that needs to be run for every potential control strategy. As in Fig. S1, note that these computation times are different from experimental values reported in the Main Text because here all validation samples are evaluated at once.

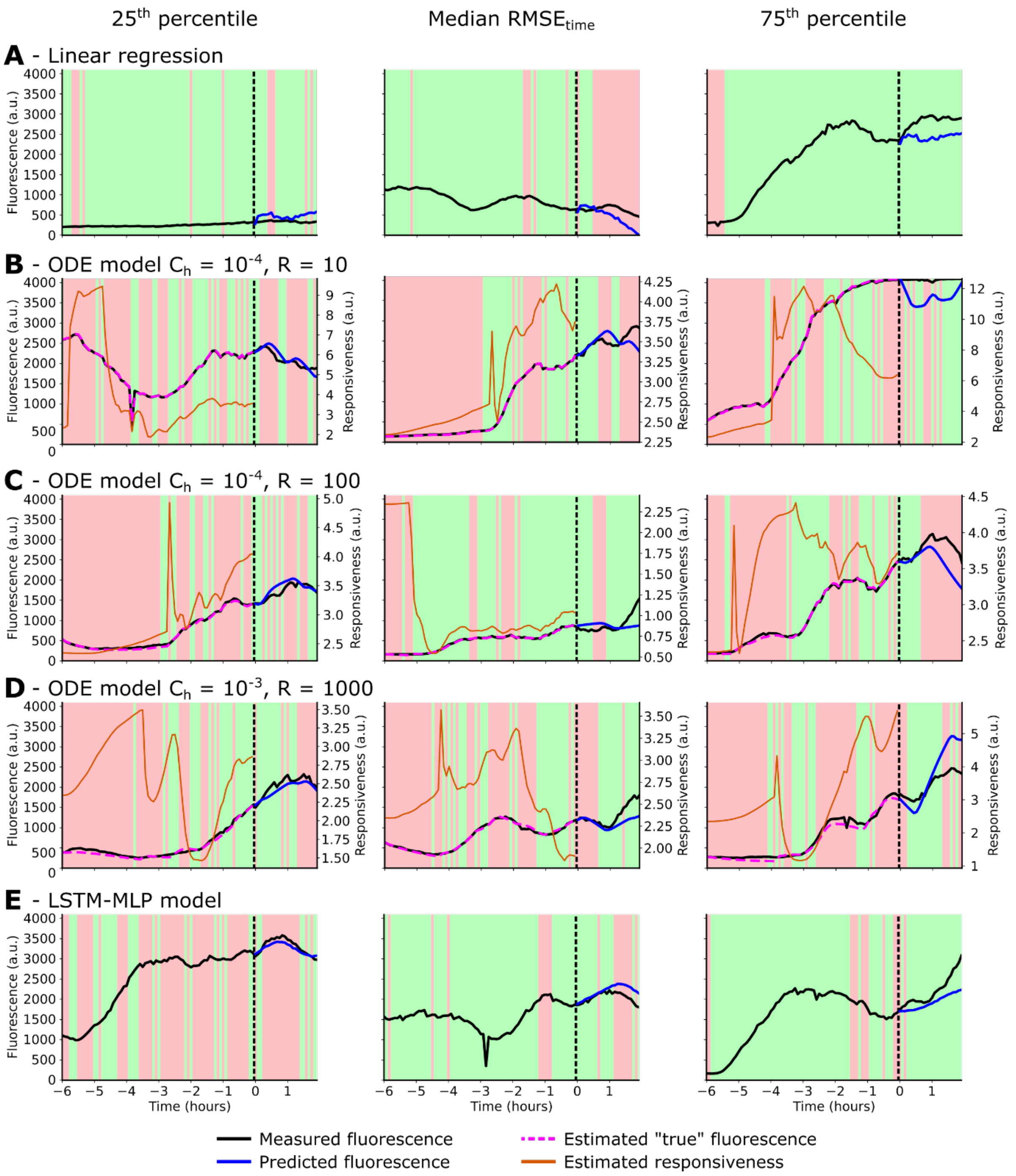

Figure S8. Representative examples of predictions for different timeseries forecasting models. For all models the leftmost column is for samples at the 25th percentile of error, the center column is for median predictions, and the rightmost column is for the 75th percentile of error. **(A)** Linear regression model. The black curve represents actual fluorescence, while the blue curve represents model prediction. **(B-D)** ODE models for different technical parameters. In addition to the black curve for measured fluorescence and the blue curve for ODE predictions, the dashed magenta curve shows estimated fluorescence levels, and the orange solid curve shows estimated responsiveness (Supplementary Text). Note that the responsiveness range changes between samples and the responsiveness values are labeled on the right side of each plot. **(E)** Our LSTM-MLP neural network model.

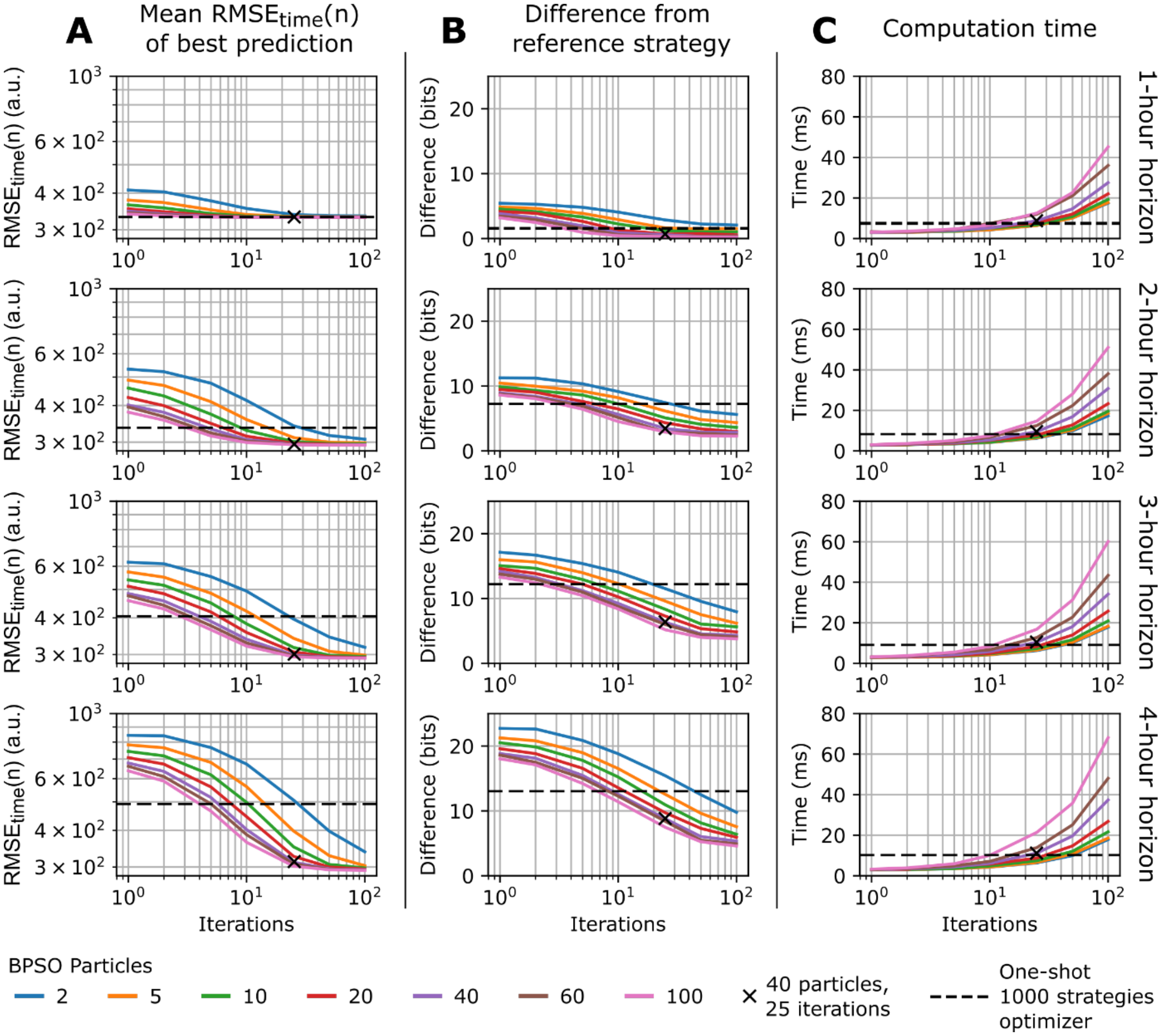

Figure S9. Binary Particle Swarm Optimizer (BPSO) evaluation for different iterations and number of particles. All evaluations were run on 524 single-cell control samples derived from experimental data. Each row from top to bottom is for the 1, 2, 3, and 4-hour horizon model. **(A)** Mean $\mathrm{RMSE}_{\mathrm{time}}(n)$ between the prediction from the "best" strategy from the BPSO and the control objective. Lower values are better, but initial transients after control is initiated set the lower limit on $RMSE_{time}(n)$, as seen with the plateau reached at higher iteration numbers. **(B)** Average difference between the "best" strategy determined by the BPSO and a reference strategy (e.g. a sequence of [red, green, red, green] and a sequence of [red, red, red, green] stimulations would differ by 1 bit). The reference strategy was determined by running the BPSO with 500 particles and 200 iterations. This represents an extreme case with many particles and a large number of iterations to identify what is essentially the optimal strategy. Differences of less than 3 bits are essentially negligible because the final points in the sequence have minimal impact on control performance. **(C)** Computation time of the BPSO for a single control sample. As in Fig. S1, note that these computation times are different from experimental values reported in the Main Text because here all samples are evaluated at once. In (A-C), we highlighted our final choice of 25 iterations and 40 particles for the BPSO (black cross). We also included the performance of a "one-shot" optimizer that selects the best out of 25×40=1000 unique potential strategies non-iteratively (black dashed line). Computation time is similar to the BPSO case with 25 iterations and 40 particles, while prediction accuracy is worse in almost all cases.

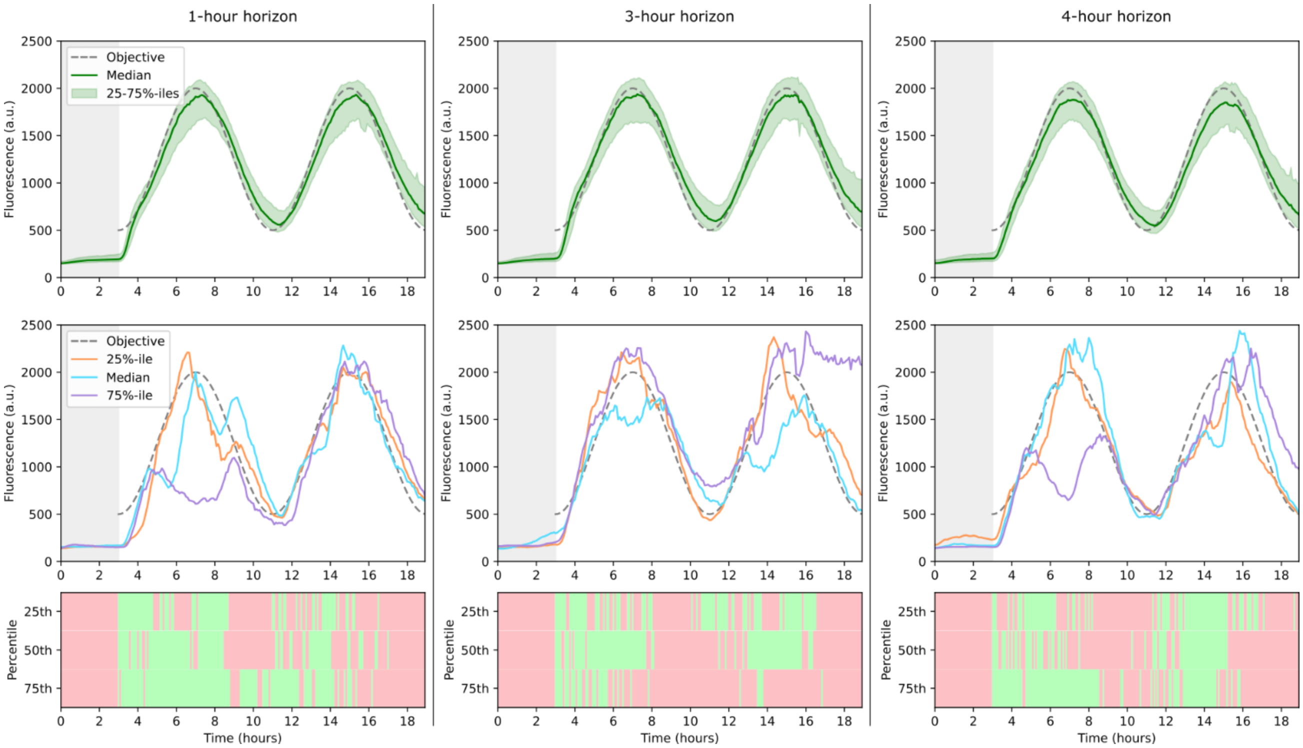

Figure S10. Control performance for different horizon controllers. 1, 3, and 4-hour horizons are shown from left to right (n = 524, 524, and 496 cells). The top row is the control performance at the population level. The gray dashed curve represents the control objective. The solid green curve represents the median fluorescence of the population. The shaded area represents 25th to 75th percentiles of the population fluorescence. The second row is the control performance at the single-cell level. Colored solid curves show representative single-cell fluorescence trajectories in the 25th, median, and 75th percentiles of control accuracy. Red and green colors in the third row represent the optogenetic stimulation sequences that were applied by the controller for the representative three single-cell trajectories, with the 25th percentile, median, and 75th percentile shown from top to bottom.

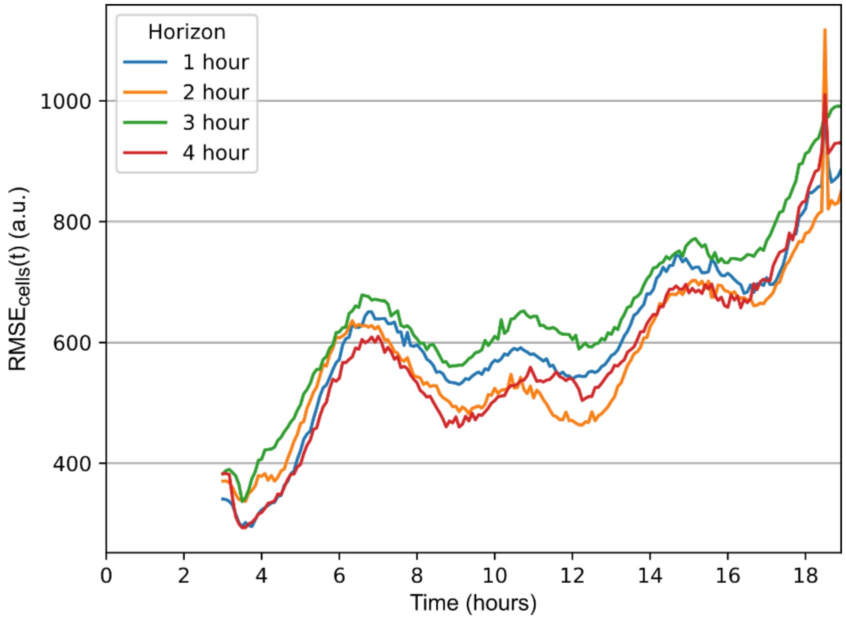

Figure S11. $\mathrm{RMSE_{cells}}(t)$ between cell fluorescence and single sinewave objective, for controllers with 1, 2, 3, and 4 hour horizons.

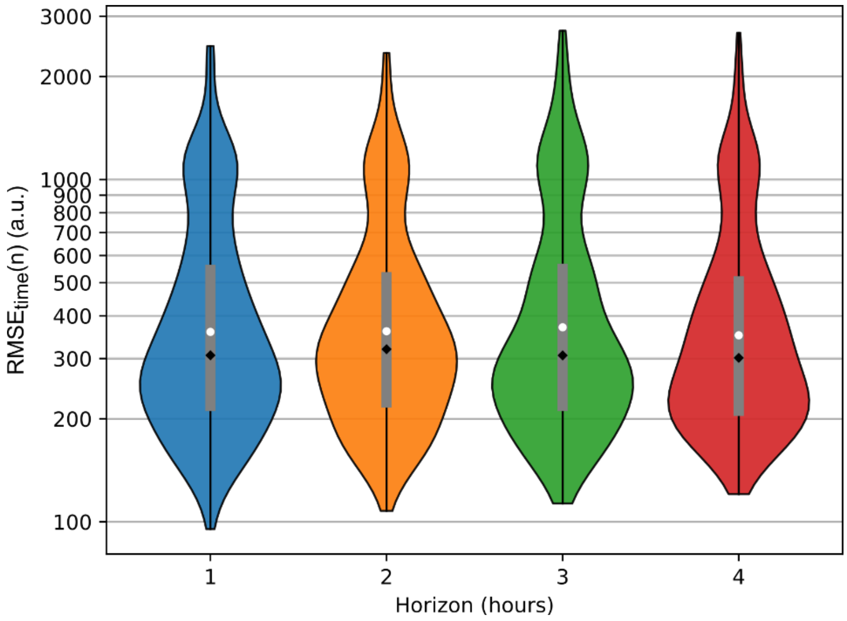

Figure S12 – Violin plot of distribution of $\mathrm{RMSE_{time}}(n)$ between single cell fluorescence and sinewave objective, for controllers with 1, 2, 3, and 4 hour horizons.

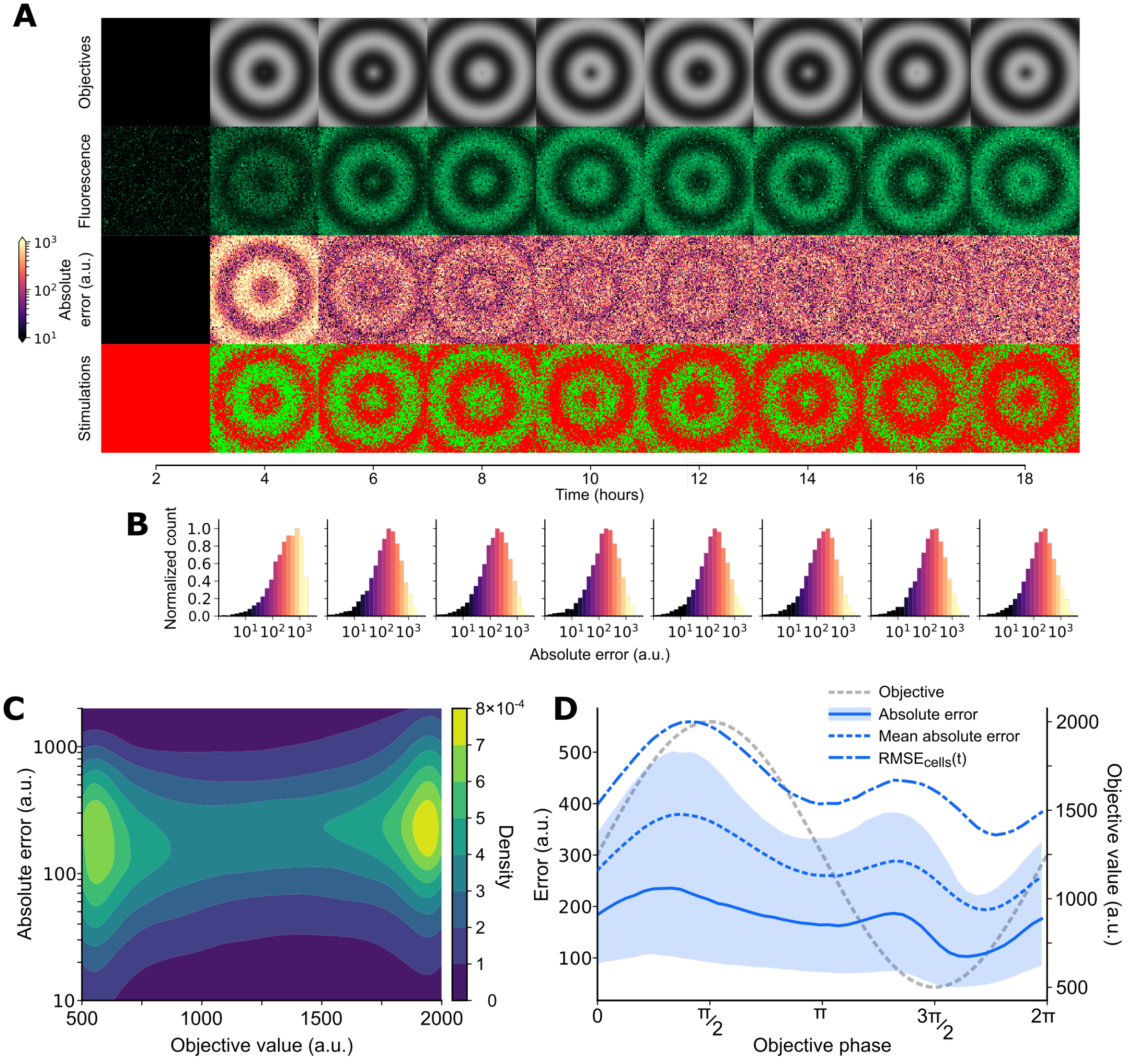

Figure S13. Error analysis for the concentric sinewaves movie. **(A)** Kymograph for the concentric movie in Fig. 3, including absolute error and optogenetic stimulations per cell. **(B)** Absolute error distribution for each kymograph timepoint. **(C)** Gaussian kernel density estimation of absolute error against objective value. As objective values increase, the distribution of error shifts up, highlighting a correlation between error and objective values. Note that the higher overall density for the lowest and highest objective values are because the sinewave objective function "spends" more time at these levels. **(D)** Error as a function of objective phase. Because all cells are subjected to the same sinewave objective with a delay, we can compile the error for different phases. Interestingly, the error is not actually minimal for exactly minimal values of the objective as panel (C) might suggest, and it seems that the controller is constrained by the dynamics of the CcaSR system and cannot perfectly track the objective trajectory as it arrives at lower objective values.

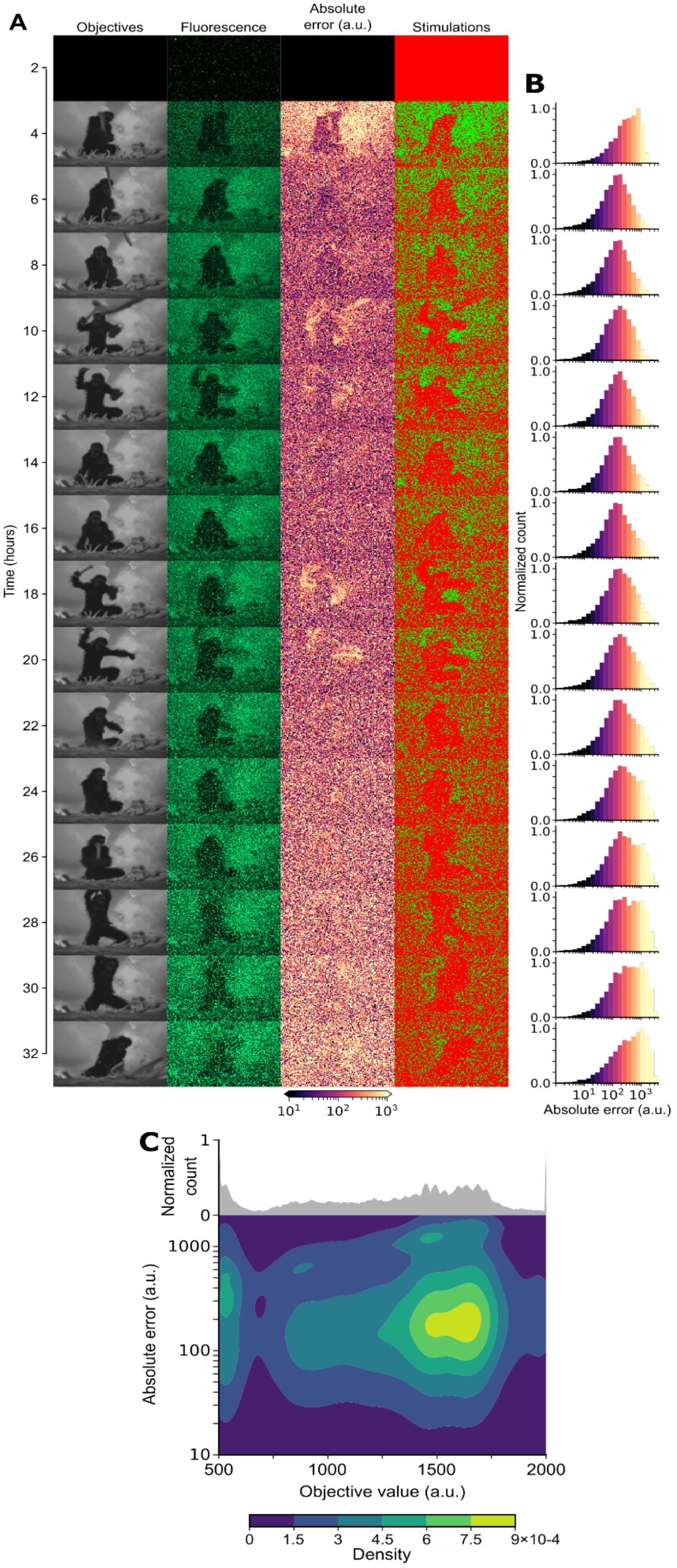

Figure S14. Error analysis for the *2001: A Space Odyssey* movie. **(A)** Kymograph for the movie in Fig. 4, including absolute error and optogenetic stimulations per cell. Rapid movement in the scene tends to cause higher error. **(B)** Absolute error distribution for each kymograph timepoint. **(C)** Gaussian kernel density estimation of absolute error against objective value. The histogram of objective values is displayed on top. Perhaps surprisingly, the distribution of error tends to shift slightly up for the lowest objective values. However, these objective values are almost entirely connected to the ape portrayed in the scene, which is responsible for most of the movement causing this slight increase in error for the lowest objective values. High objective values also produce higher errors, as the controller struggles to maintain cells at these extreme levels.

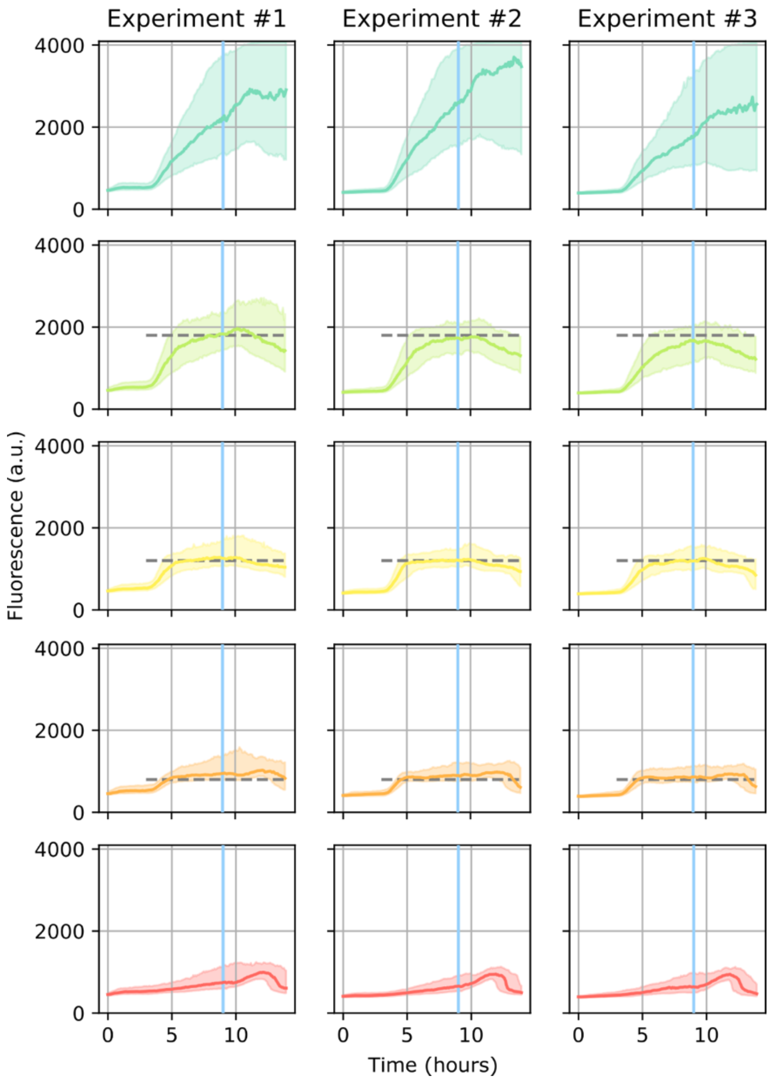

Figure S15. Population response with *tetA-gfp* control per category and replicate experiment. Top and bottom row represent the cells subjected to constant green and constant red optogenetic stimulations, respectively. The middle rows represent cells controlled at 1800, 1200, and 800 units of fluorescence. All cells received exclusively red stimulations for equilibration until t = 3h. Horizontal dashed gray lines represent control objectives. Vertical blue lines represent the time of tetracycline addition at t = 9h. Solid colored curves represent the population median fluorescence. The shaded areas represent the 25th to 75th fluorescence percentiles.

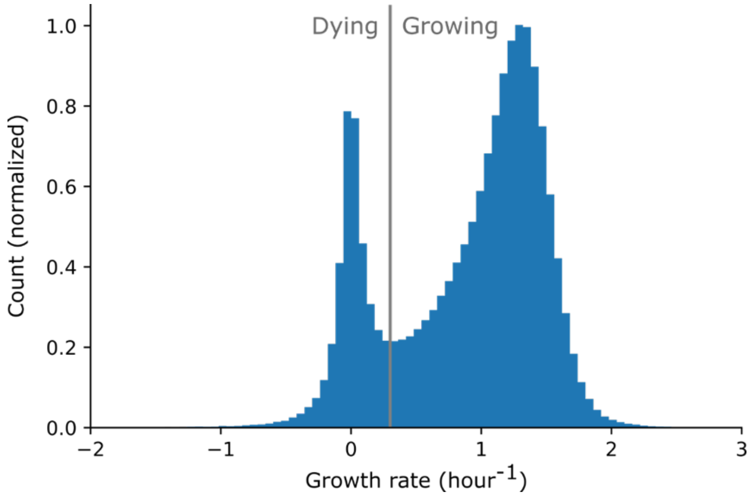

Figure S16. Histogram of growth rates for *tetA-gfp* control experiments, across all replicates, cell populations, and timepoints. Single-cell growth rates were smoothed with a median filter sliding over a one-hour time window. The vertical gray line represents the 0.3 hour^{-1} "growing" vs. "dying" threshold.

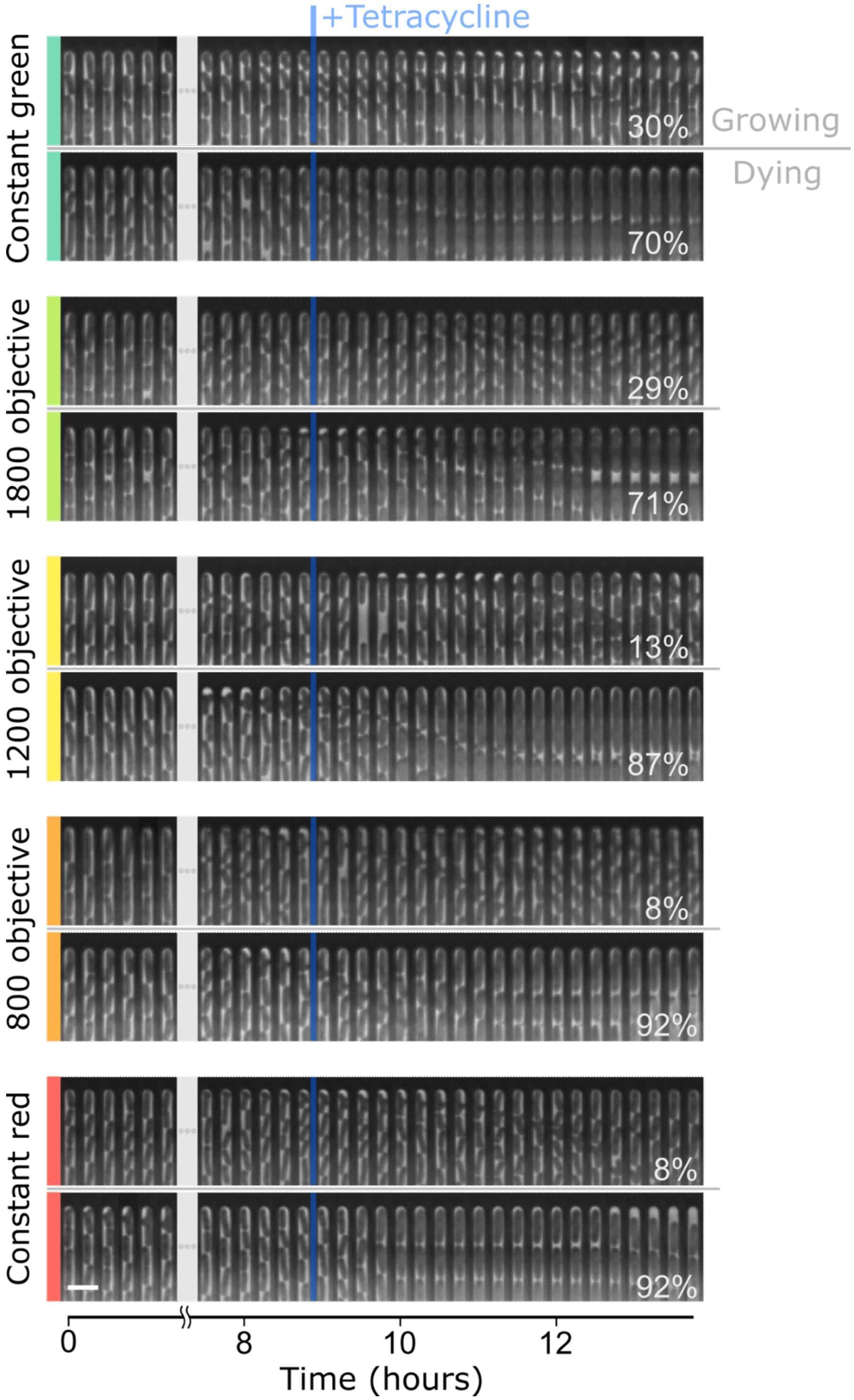

Figure S17. Representative kymographs of cells in the "growing" or "dying" sub-populations for each category of cells. Listed values indicate percentage of cells in each sub-population at the end of the experiment. Scale bar, 5 µm.

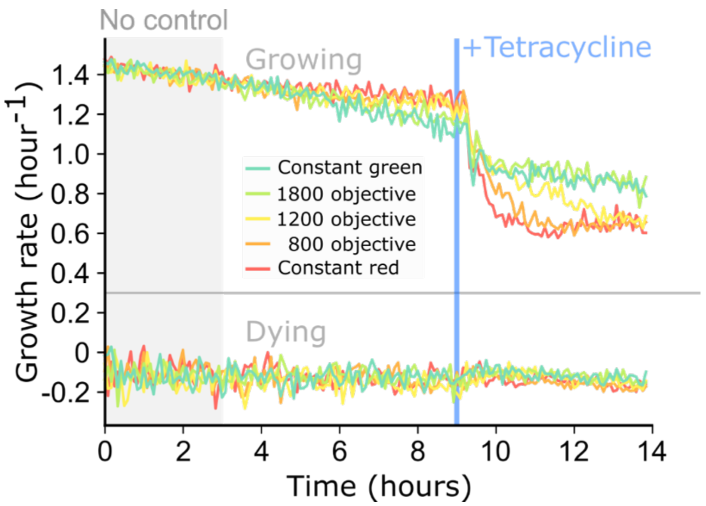

Figure S18. Median growth rates over time for cells in the “growing” or the “dying” sub-populations. Data from all replicates were merged. The growth rate over time was not smoothed.

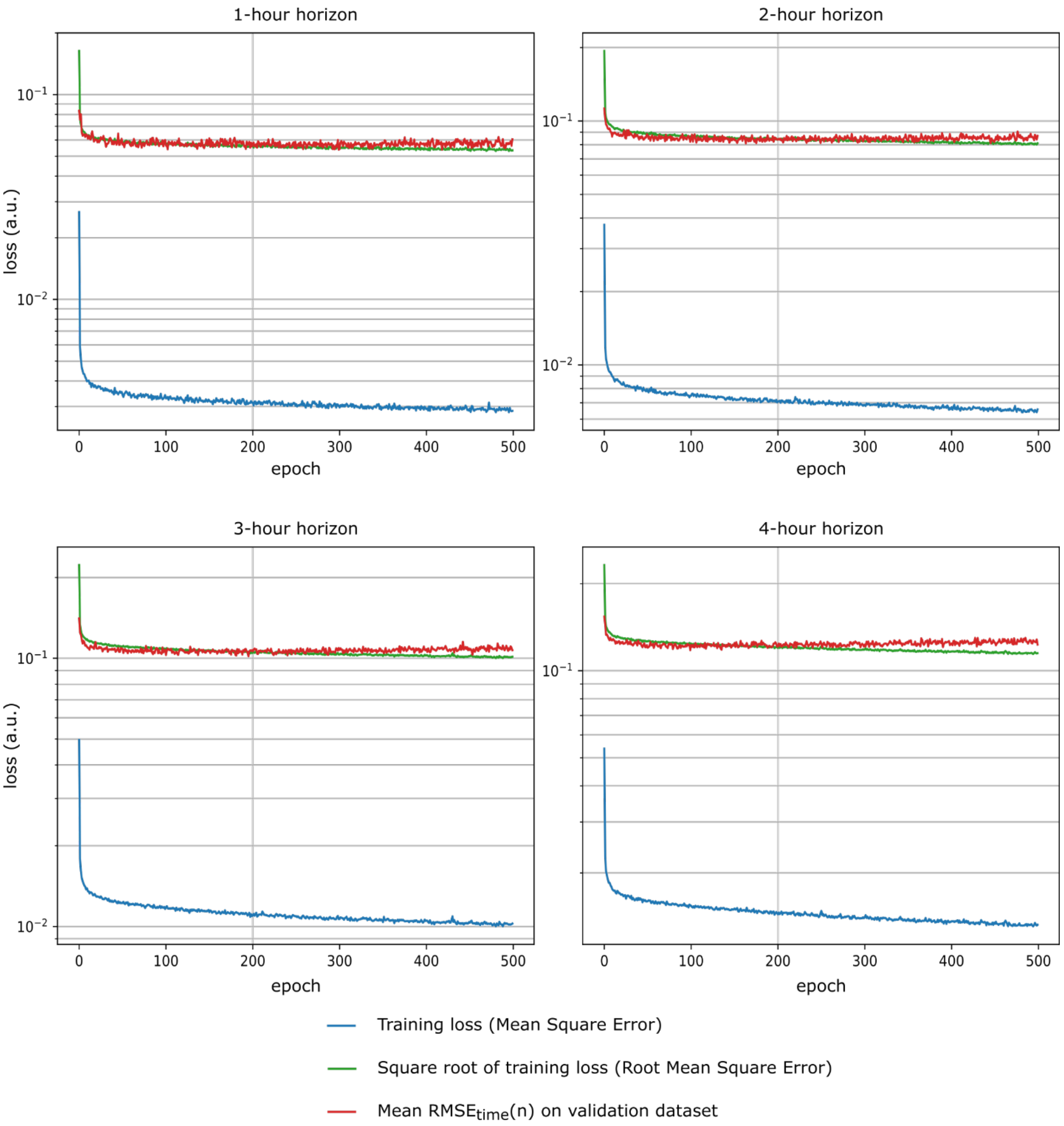

Figure S19. Training loss and validation error of the 1, 2, 3, and 4-hour horizon models. The models were trained for up to 500 epochs. At the end of each epoch, the model was evaluated against 10,000 samples from the validation dataset. For all horizons, we observed that around 200 epochs the validation error seemed to plateau or even increase while the training loss kept decreasing.

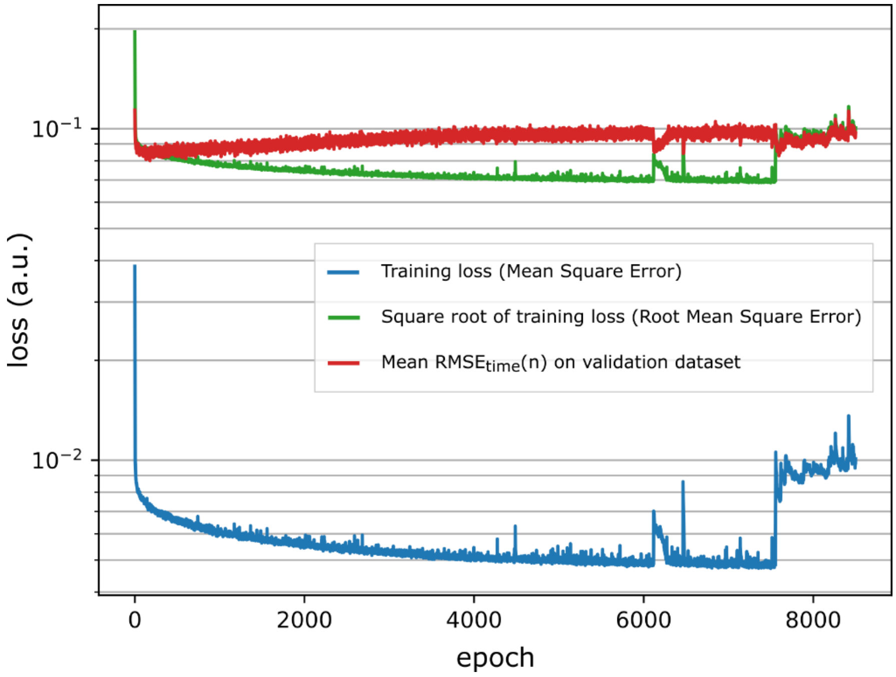

Figure S20. Training loss and validation error of the 2-hour horizon model up to 8,504 epochs. The maximum number of epochs was set to 10,000, with a patience parameter that stopped the training 1,000 epochs after the training loss stopped decreasing. Validation error increases as loss decreases, indicating model overfitting. The red curve represents the mean $\mathrm{RMSE}_{\mathrm{time}}(n)$ over the validation dataset at each epoch.

	½ hour	1 hour	2 hours	3 hours	4 hours	6 hours	8 hours	10 hours	12 hours
**Median $\mathbf{RMSE_{time}}(\boldsymbol{n})$**	287	267	262	259	260	259	264	267	272

Table S1. Median $\mathrm{RMSE_{time}}(n)$ of predictions from the linear regression model for different past input lengths.

	$\boldsymbol{a}$	$\boldsymbol{b}$	$\boldsymbol{c_2}$	$\boldsymbol{\kappa}$	$\boldsymbol{n_h}$	$\boldsymbol{\tau}$	$\boldsymbol{h_1}$	$\boldsymbol{h_2}$
**Initial values**	22.6	0.0104	0.0631	0.485	3.67	12.0	0.0710	0.0303
**Fitted values**	25.4	0.00905	0.120	1.70	1.01	10.0	0.0710	0.0303

Table S2. Parameter values for the ODE model, before and after fitting to our data. The initial values were taken from Chait et al[1]. For the fitted values, we fitted all parameters to our data except for h_1 and h_2 (Supplementary Text). Note that the values for h_1 and h_2 that were actually used during model evaluation were multiplied by the responsiveness dynamics coefficient C_h.

Responsiveness dynamics $\mathbf{C_h}$ (columns); Technical noise variance $\mathbf{R}$ (rows)

	1e-06	1e-05	1e-04	1e-03	1e-02	1e-01	1e+00	1e+01	1e+02	1e+03
1e-02	333	333	320	346	458	467	475	406	405	406
1e-01	331	331	319	347	458	466	476	405	405	406
1e+00	329	331	320	343	462	462	475	405	405	406
1e+01	326	329	319	340	460	472	474	405	405	406
1e+02	322	324	311	339	438	437	462	400	406	408
1e+03	338	342	331	331	407	411	451	403	408	409
1e+04	401	399	392	379	383	425	424	411	426	428
1e+05	458	457	450	423	385	389	407	436	443	443
1e+06	472	472	472	458	412	402	431	478	480	480
1e+07	499	499	497	496	469	453	514	549	554	555
1e+08	630	630	629	624	614	563	651	675	679	679

Table S3. Median $\mathrm{RMSE_{time}}(n)$ of ODE-based predictions with initial parameter values for different values of filtering parameters C_h and R.

Responsiveness dynamics $\mathbf{C_h}$ (columns); Technical noise variance $\mathbf{R}$ (rows)

	1e-06	1e-05	1e-04	1e-03	1e-02	1e-01	1e+00	1e+01	1e+02	1e+03
1e-02	278	274	254	267	374	356	386	386	375	373
1e-01	273	272	253	267	381	355	386	386	375	373
1e+00	270	270	253	267	376	352	386	385	373	372
1e+01	259	258	244	268	372	350	388	384	372	372
1e+02	258	255	245	259	328	336	382	386	373	374
1e+03	268	265	253	249	314	333	381	383	381	381
1e+04	309	310	295	270	284	330	372	384	387	386
1e+05	343	343	331	307	284	284	345	391	392	392
1e+06	357	357	356	346	309	298	375	413	415	416
1e+07	399	399	398	394	373	352	464	486	489	489
1e+08	577	577	577	576	551	508	590	607	610	611

Table S4. Median $\mathrm{RMSE_{time}}(n)$ of ODE-based predictions with fitted parameter values for different values of filtering parameters C_h and R. The three red contoured values highlight the 3 combinations of C_h and R that minimize prediction error.

Supplementary Text

Deep model predictive control

For a specific cell, we define the set of measurements at timepoint t, $\mathrm{X}_t = \left\{x_t^{(f)}\right\} \in \mathbb{R}^7$ with f in the set F of the measured single-cell features {fluorescence, cell area, cells in chamber, mean chamber fluorescence, standard deviation of chamber fluorescence, neighbor stimulations, image sharpness}. The system inputs $l_t \in \{0,1\}$ denote whether a red light stimulation (0) or a green light stimulation (1) is applied to the cell at timepoint t. We define $\mathrm{X}_{t_1:t_2}$ as the sequence of measurements from time t_1 through time t_2, and $\mathrm{L}_{t_1:t_2}$ is similarly defined for system inputs. Finally for clarity we define $\mathrm{Y}_{t_1:t_2} = \{y_t | t \in [\![t_1, t_2]\!]\} = \left\{x_t^{\text{fluorescence}} \middle| t \in [\![t_1, t_2]\!]\right\}$, the sequence of measurements for only fluorescence from time t_1 through time t_2.

Our approach is based on the model predictive control framework with a receding horizon, which can be seen as solving, once single cell measurements have been acquired at each time point t, an open-loop optimization problem over a finite prediction horizon τ. In our case, given a function ψ_k that can predict future fluorescence values $\hat{y}_k = \psi_k(X_{0:t}, L_{0:t}, L_{t+1:t+\tau})$ with $k \in [\![t+1, t+\tau]\!]$, and an arbitrary objective sequence $\mathrm{O}_{t+1:t+\tau} = \{o_k | k \in [\![t+1, t+\tau]\!]\} \in \mathbb{R}^\tau$ that we want our cell's fluorescence to follow, the goal at each new timepoint *t* is to find the optimal light inputs sequence $\mathrm{L}^*_{t+1:t+\tau}$ such that:

$$\mathrm{L}^*_{t+1:t+\tau} = \underset{\mathrm{L}_{t+1:t+\tau}}{\arg\min} f(\mathrm{L}_{t+1:t+\tau}), \qquad \text{with } f(\mathrm{L}_{t+1:t+\tau}) = \sqrt{\frac{1}{\tau}\sum_{k=t+1}^{t+\tau} \left(\psi_k(\mathrm{X}_{0:t}, \mathrm{L}_{0:t}, \mathrm{L}_{t+1:t+\tau}) - o_k\right)^2} \quad (1)$$

Once this optimal strategy is identified, l^*_{t+1} is applied to the cell. After the sampling time of 5 minutes has elapsed, X_{t+1} is acquired, and the optimization problem in Equation 1 is solved again given the new timepoint and data. Note that under this convention and sampling rate, l_{t+1} is closer in time to X_t than to X_{t+1}.

The innovation of deep model predictive control is to establish a neural network function ψ_k^{W} to predict fluorescence at a future timepoint $\hat{y}_k = \psi_k^{\mathrm{W}}(\mathrm{X}_{0:t}, \mathrm{L}_{0:t}, \mathrm{L}_{t+1:t+\tau})$ where $\mathrm{W} \in \mathbb{R}^\omega$ denotes parameters of the function (i.e. the ω weights and biases of the neural network). The optimal parameters W^* are "learned" such that they minimize the distance between predicted fluorescence and subsequent measurements for randomly sampled past timepoints and across all cells. The loss function that our training routine tries to minimize is the following for a single cell and timepoint t:

$$\frac{1}{\tau}\sum_{k=t+1}^{t+\tau} \left(\psi_k^{\mathrm{W}}(\mathrm{X}_{0:t}, \mathrm{L}_{0:t}, \mathrm{L}_{t+1:t+\tau}) - y_k\right)^2 \quad (2)$$

In practice, learning is actually done over so-called batches of training samples that group together several random timepoints and different cells.

Also note that this framework permits online learning, where the parameters are re-optimized on-the-fly as new data are acquired during control experiments. While this is an interesting avenue for future research, for the sake of simplicity and to ensure stable control throughput, as well as to allow us to investigate function accuracy beforehand, in this study we first acquire training datasets offline to learn the set of optimal parameters W^* prior to running model predictive control. These data were acquired by

subjecting cells to random optogenetic stimulations (see below) and using these measurements as reference for training. The fitted network parameters W^* were thus fixed during control experiments.

Deep neural networks have proven to be excellent at learning complex functions and accurately forecasting timeseries like ours, and they can be implemented to run efficiently on modern computer hardware. However, the number of possible light sequences to consider as potential control inputs grows exponentially with τ, and for this reason we had to implement an optimizer to efficiently reach an optimal or near-optimal solution for Equation. 1.

Binary Particle Swarm Optimizer

Particle swarm optimization (PSO) evaluates a number of potential solutions in parallel (particles) over a certain number of iterations and selects the best solution. The binary particle swarm optimization algorithm (BPSO) is a variant of a traditional PSO that operates in a binary search space[2,3], on pseudo-Boolean functions $\{0,1\}^m \rightarrow \mathbb{R}$. In our case, the function we want to minimize is $f: \{0,1\}^\tau \rightarrow \mathbb{R}_0^+$ from Equation 1. The key difference compared to a traditional PSO is that both particle velocity and position are updated probabilistically, as positions cannot change gradually.

Each particle n consists of a maintained 3-tuple $(\mathrm{L}^{(n)}, \mathrm{L}^{(n)*}, v^{(n)})$ where $\mathrm{L}^{(n)} \epsilon \{0,1\}^\tau$ is the particle's current position in the search space (i.e. in our case, a possible future light input sequence $\mathrm{L}_{t+1:t+\tau}$, written L for clarity), $\mathrm{L}^{(n)*} \epsilon \{0,1\}^\tau$ is the particle's best position so far, and $v^{(n)} \epsilon \mathbb{R}^\tau$ is the particle velocity in the space. We also define L^{G*}, the global best position/sequence so far across all particles, and $r^{cog}, r^{soc} \epsilon (U[0,2])^\tau$ two uniformly random vectors that will be sampled at each iteration to update velocities (the so-called "cognitive" vector used to update velocities on the basis of the particle's own best position so far, and the "social" vector for the best position so far across all particles). Finally, we define the sigmoid function $s(v) = 1/(1+e^{-v})$. The algorithm below illustrates our implementation of the BPSO. We consider a total of π particles and i^{max} iterations.

Algorithm 1 (Binary Particle Swarm Optimization)

1. Initialize velocities with $v_i := 0^\tau$
   Set iterations counter $i := 0$
2. Sample r^{cog} and r^{soc}
   Set $i := i + 1$
3. For $n :=$ 1 to π do:
   For $k :=$ 1 to τ do:
   Set $l_k^{(n)} := 1$ with probability $s\left(v_k^{(n)}\right)$; otherwise $l_k^{(n)} := 0$
4. For $n :=$ 1 to π do:
   If $f\left(\mathrm{L}^{(n)}\right) < f\left(\mathrm{L}^{(n)*}\right)$ or $i = 1$ then $\mathrm{L}^{(n)*} := \mathrm{L}^{(n)}$
   If $f\left(\mathrm{L}^{(n)*}\right) < f(\mathrm{L}^{G*})$ or ($i = 1$ and $n = 1$) then $\mathrm{L}^{G*} := \mathrm{L}^{(n)*}$
5. For $n :=$ 1 to π do:
   For k := 1 to τ do:
   $v_k^{(n)} := v_k^{(n)} + r_k^{cog} \cdot (l_k^{(n)*} - l_k^{(n)}) + r_k^{soc} \cdot (l_k^{G*} - l_k^{(n)})$
   Set $v_k^{(n)} := \max\{v_k^{(n)}, -4\}$
   Set $v_k^{(n)} := \min\{v_k^{(n)}, +4\}$
6. If $i = i^{max}$ stop; otherwise go to 2

At step 3, particle positions, i.e. light input sequences, are updated probabilistically based on the sigmoid of the velocities. At step 4, function f is evaluated on all the particles positions, and then the best positions are updated. At step 5, based on the new best positions for each particle and the global best position, velocities are updated probabilistically, and their values clipped to $[-4, +4]$. Finally, once the maximum number of iterations is reached, the best light sequence L^{G*} is returned. For more details on this implementation see Sudholt et al. [3].

Random optogenetic stimulation sequences for training set experiments

For our training sets, we want the pre-determined optogenetic stimulations sequences to be as diversified as possible, but also for the sequences to often contain relatively long sub-sequences with only red or green stimulations. Simply generating a Bernoulli sequence of random coin flips, even biased, would rapidly alternate between red and green stimulations for most of the sequence. Instead, we used a bounded one-dimensional random walk to generate our training stimulation sequences:

1. For each cell, a uniformly random sequence of either +1 or -1 values was generated. The sequence was generated for up to 36 hours of stimulations every 5 minutes, or 432 total values, although we only ended up running training experiments for up to 24 hours. An example sequence would be:

   -1, -1, 1, -1, -1, -1, -1, -1, 1, 1, -1, 1, -1, -1, -1, 1, 1, 1, 1, 1, -1, 1, -1, 1, -1, -1, 1, 1, -1, …

2. This random sequence was then cumulatively summed over time, however that sum was bounded to maximum and minimum values, respectively positive and negative. This prevents cumulatively summed values from growing too large or too small. Different sets of maximum and minimum values between +4 and -3 were used randomly for different cells, to generate different distributions of red and green stimulations. The cumulative sum of the sequence above bounded to [-2, 2] would be:

   0, -1, -2, -1, -2, -2, -2, -2, -2, -1, 0, -1, 0, -1, -2, -2, -1, 0, 1, 2, 2, 1, 2, 1, 2, 1, 0, 1, 2, …

3. Finally, the values are binarized. All values greater than or equal to 0 are set as green stimulations while negative values are set to red stimulations. The final sequence for the example would be: G, R, R, R, R, R, R, R, R, R, G, R, G, R, R, R, R, G, G, G, G, G, G, G, G, G, G, G, G, G, …

At the beginning of each sequence we inserted 3 hours (=36 time points) of only red stimulations to mimic control conditions and make sure cells started in a repressed state.

Single-cell feature normalization for neural network processing

Input normalization is good practice for training and using neural networks. We used the following formulas to ensure single-cell timeseries data were consistently normalized to the same range of values:

- **Fluorescence**: Single-cell fluorescence measurements were limited to [0, 4095], the dynamic range of the data. Fluorescence feature values (mother cell fluorescence, average chamber fluorescence, and chamber fluorescence standard deviation) were thus simply normalized linearly:

$$x_{norm} = x_{raw}/4095$$

  Where x_{raw} is the initial measured value for a given feature and time point, and x_{norm} is the

resulting normalized value that is used as input for the neural network, both for training and on-the-fly for feedback control.

- **Cell area:** Mother cell area, measured in pixels, theoretically has no clear upper bound. After analyzing area distributions, we used a negative exponential function:

$$x_{norm} = 1 - 10^{-x_{raw}/3000}$$

- **Chamber cell count**: The number of cells in the chamber at any given timepoint also theoretically has no clear upper bound. We also used a negative exponential function with a different normalization factor:

$$x_{norm} = 1 - 10^{-x_{raw}/9}$$

- **Chamber image sharpness**: Image sharpness, measured as the average of the Laplacian of the image, has no clear upper or lower bound. We used the following formula to normalize sharpness to [0, 1]:

$$x_{norm} = \frac{1}{1 + e^{-x_{raw}+3.6}}$$

Linear regression

We used the Tensorflow library to implement the linear regression model. We implemented a neural network with a single layer of fully-connected neurons with linear activation, which is mathematically equivalent to a linear regression model. The number of neurons in that single layer, and therefore the length of the output vector, was set to $\tau = 24$, the number of timepoints for our 2-hour prediction horizon. This architecture, however, cannot handle input timeseries of varying length, unlike LSTM networks. For this reason, we evaluated a range of past input lengths ϕ ranging from 6 to 144 timepoints, corresponding to 0.5 to 12 hours. All 7 measured features in set F from the past timeseries were flattened and concatenated with the light stimulation vectors, resulting in input vector lengths ranging from 72 to 1176. Mathematically, this implementation is equivalent to the following formula for ψ_k^{W}, with $k \in [\![t+1, t+\tau]\!]$:

$$\psi_k^{\mathrm{W}}(\mathrm{X}_{t-\phi:t}, \mathrm{L}_{t-\phi:t}, \mathrm{L}_{t+1:t+\tau}) = \sum_{f}^{\mathrm{F}} \sum_{p=t-\phi}^{t} \alpha_{kp}^{(f)} \cdot x_p^{(f)} + \sum_{p=t-\phi}^{t+\tau} \alpha_{kp}^{'} \cdot l_p + \beta_k \quad (3)$$

with:

- $\alpha_{kp}^{(f)}$ the regression coefficient for measured feature f, past timepoint p, and future timepoint k
- $x_p^{(f)}$ the measured value of feature f at past timepoint p
- α_{kp}' the regression coefficient for the system inputs for timepoint p and future timepoint k
- l_p the system inputs, or light stimulations, at timepoint p
- β_k the intercept term for future timepoint k

Note that in this case, the set of parameters W becomes the collection of all coefficients and intercept terms $\alpha_{kp}^{(f)}$, α'_{kp}, and β_k. Because the only difference in terms of implementation between this linear regression model and our encoder-decoder model was the network architecture, we were able to re-use the same training procedure as described in the Methods section and Equation 2 to fit the regression coefficients and the intercept terms to our data by computing the mean square error between the vector of fluorescence predictions against the measured fluorescence data (Table S1). Past input lengths ϕ of 36, 48, and 72, (or 3, 4, and 6 hours) were similarly accurate and we picked the 6 hours past length model for comparison with our LSTM-MLP model and with the ODE-based model (Fig. S7).

Differential equation model

To compare our model with the state of the art, we used the same model as described by Chait et al.[1] to predict the response of our system to optogenetic inputs. The genetic system in their study is very similar to the one we use in most of this study, but we use a more recent version of the CcaSR system[4], a different fluorescent reporter, a different *E. coli* strain, a shorter sampling time, and of course entirely different microscopy hardware. We therefore used the same set of ordinary differential equations (ODEs) but fitted its parameters to our data. The system of ODEs below is copied from their study; note that variables and parameters names in this section and the next are different from the rest of the Supplementary Text to maintain consistency with the notation of Chait et al.[1]:

$$\frac{\partial}{\partial t}H(t) = u(t-\tau_{delay}) - c_2 \cdot H(t) \quad (4)$$

$$\frac{\partial}{\partial t}\mathbb{E}[E(t)] = h_1 - h_2 \cdot \mathbb{E}[E(t)] \quad (5)$$

$$\frac{\partial}{\partial t}\mathbb{E}[F(t)] = a \cdot L(t) \cdot \mathbb{E}[E(t)] - b \cdot \mathbb{E}[F(t)] \quad (6)$$

$$\frac{\partial}{\partial t}\mathbb{E}[E(t)^2] = h_1 + 2h_1 \cdot \mathbb{E}[E(t)] + h_2 \cdot \mathbb{E}[E(t)] - 2h_2 \cdot \mathbb{E}[E(t)^2] \quad (7)$$

$$\frac{\partial}{\partial t}\mathbb{E}[E(t)F(t)] = h_1 \cdot \mathbb{E}[F(t)] + a \cdot L(t) \cdot \mathbb{E}[E(t)^2] - (h_2 + b) \cdot \mathbb{E}[E(t)F(t)] \quad (8)$$

$$\frac{\partial}{\partial t}\mathbb{E}[F(t)^2] = b \cdot \mathbb{E}[F(t)] + a \cdot L(t) \cdot \mathbb{E}[E(t)] + 2a \cdot L(t) \cdot \mathbb{E}[E(t)F(t)] - 2b \cdot \mathbb{E}[F(t)^2] \quad (9)$$

with $L(t) = \frac{(H(t) \cdot c_2)^{n_H}}{\kappa + (H(t) \cdot c_2)^{n_H}}$

Variable *H(t)* represents the activation dynamics of the CcaSR system and incorporates a time delay on the optogenetic stimulations $u(t)$. *E(t)* is a stochastic variable that represents the cell-to-cell variability in the "responsiveness" of the system. *F(t)* is the measured fluorescence and is also a stochastic variable. Equations 4-6 define the dynamics of the first order moments of the system that will be used to predict single-cell response to optogenetic stimulations. Additionally, Equations 7-9 describe the dynamics of the second order moments that are necessary for state estimation (see below).

To fit the model parameters to our data, we used the same training data as we used to train our neural network model. Because all single-cell traces in the training sets respond to different optogenetic stimulation sequences, we could not derive second order statistics from our data, and therefore could only use Equations 4-6 to fit model parameters. Because of this, parameters h_1 and h_2 that govern the dynamics of cell-to-cell variability were not identifiable. We circumvented this issue by fixing the value of h_1 and h_2 during fitting. Later we evaluated prediction accuracy after multiplying both of them by the same factor C_h ranging from 10^{-6} to 10^3 (Table S3, S4). Doing so maintains the steady state values for the

first and second order moments of $E(t)$ and only changes how fast the responsiveness is estimated to change. We note that Chait and colleagues faced a similar issue[1], and opted to fix h_1 and consider h_2 a filtering parameter to tune after fitting.

We used the covariance matrix adaptation evolution strategy[5] to fit model parameters to the data. Because we have such large training sets and we are fitting to single-cell traces, evaluating the ODE model against all training samples would have been extremely slow from a computational perspective. Instead, at every iteration we randomly picked 100 single-cell trajectories and compared the predicted fluorescence response from the ODE model to the measured single-cell response. The function output to minimize was the average $\mathrm{RMSE}_{\mathrm{time}}(n)$ between model prediction and measured response for fluorescence across all 100 trajectories. We used the parameters from Chait et al. as initial values, except for parameter a, which we multiplied by 80 to roughly match the dynamic range of our measurements. The fit converged to the parameter values listed in Table S2.

State estimation

In order to predict future fluorescence responses, the state of the system needs to be estimated from past measurements. We used the same hybrid Kalman filter approach as described by Chait et al. Briefly, the state estimator separates technical measurement noise from fluorescence fluctuations caused by gene expression variability and estimates the true fluorescence level (F), cell responsiveness (E), and CcaSR activation (H) level at each time point by comparing model predictions to measurements. Technical noise is assumed to be additive, time-invariant Gaussian noise with mean zero and variance R. Because the prior for the covariance matrix of the system can be calculated with Equations 7-9, we can estimate how much of the fluctuations in measured fluorescence levels can be attributed to noise or to actual changes in the system's state. By tuning the value for R, we can adjust the amount of noise rejection from the filter, i.e. how much the filter should "trust" the model vs. measurements. By changing the value for C_h, and therefore h_1 and h_2, we tune the rate at which the value of E can change according to the model, and therefore how much apparent rapid changes in responsiveness will be considered noise by the filter. We refer the reader to Chait et al.[1] for a detailed mathematical description of the state estimator algorithm, and to our code repository for a Python implementation of that algorithm.

For evaluation, the data was structured in the same way as for other models, with prediction inputs being past fluorescence and past optogenetic stimulations combined with future optogenetic stimulations, and where the output is the predicted fluorescence response that is compared to the measured future fluorescence. For every sample, state estimation was run over past fluorescence measurements and stimulations to estimate the system's state, including hidden variables (H and E), up to "present". In order to compare results to the linear regression model, we set the amount of past measurements to 6 hours, however like our neural network the Kalman filter can be run on any amount of past data. From the estimated "present" state, 2 hours of future system evolution were predicted, and the $\mathrm{RMSE}_{\mathrm{time}}(n)$ between predicted fluorescence and measured fluorescence over the prediction horizon was computed. To first determine optimal values of R and C_h, we evaluated the error over a sub-set of 1000 samples in the evaluation set for both the original non-fitted parameters and our fitted parameters (Tables S3, S4). Then we selected the best 3 combinations of R and C_h to evaluate against the rest of our validation set, and compared those results to the performance of our encoder-decoder model and the linear regression model both in terms of prediction accuracy and computation time (Fig. S7, S8).

Supplementary References

1. Chait, R., Ruess, J., Bergmiller, T., Tkačik, G. & Guet, C. C. Shaping bacterial population behavior through computer-interfaced control of individual cells. *Nat Commun* **8**, 1535 (2017).

2. Kennedy, J. & Eberhart, R. C. Discrete binary version of the particle swarm algorithm. *Proceedings of the IEEE International Conference on Systems, Man and Cybernetics* **5**, 4104–4108 (1997).

3. Sudholt, D. & Witt, C. Runtime analysis of a binary particle swarm optimizer. *Theor Comput Sci* **411**, 2084–2100 (2010).

4. Ong, N. T. X. & Tabor, J. J. A miniaturized E. coli green light sensor with high dynamic range. *ChemBioChem* (2018) doi:10.1002/cbic.201800007.

5. Hansen, N. & Ostermeier, A. Adapting arbitrary normal mutation distributions in evolution strategies: the covariance matrix adaptation. in *Proceedings of IEEE International Conference on Evolutionary Computation* 312–317 (IEEE, 1996). doi:10.1109/ICEC.1996.542381.
