# Supplementary material for: Deep model predictive control of gene expression in thousands of single cells
Source: Nat Commun. 2024 Mar 8;15:2148. doi: 10.1038/s41467-024-46361-1 (PMC10923782; doi:10.1038/s41467-024-46361-1)
Supplement: Supplementary file 2 — Reporting Summary [file 41467_2024_46361_MOESM2_ESM.pdf]

nature portfolio

Corresponding author(s): Mary J. Dunlop
Jean-Baptiste Lugagne

Last updated by author(s): Sep 22, 2023

Reporting Summary

Nature Portfolio wishes to improve the reproducibility of the work that we publish. This form provides structure for consistency and transparency in reporting. For further information on Nature Portfolio policies, see our Editorial Policies and the Editorial Policy Checklist.

Statistics

For all statistical analyses, confirm that the following items are present in the figure legend, table legend, main text, or Methods section.

n/a	Confirmed	
☐	☒	The exact sample size (*n*) for each experimental group/condition, given as a discrete number and unit of measurement
☐	☒	A statement on whether measurements were taken from distinct samples or whether the same sample was measured repeatedly
☒	☐	The statistical test(s) used AND whether they are one- or two-sided<br>*Only common tests should be described solely by name; describe more complex techniques in the Methods section.*
☒	☐	A description of all covariates tested
☒	☐	A description of any assumptions or corrections, such as tests of normality and adjustment for multiple comparisons
☐	☒	A full description of the statistical parameters including central tendency (e.g. means) or other basic estimates (e.g. regression coefficient) AND variation (e.g. standard deviation) or associated estimates of uncertainty (e.g. confidence intervals)
☒	☐	For null hypothesis testing, the test statistic (e.g. *F*, *t*, *r*) with confidence intervals, effect sizes, degrees of freedom and *P* value noted<br>*Give P values as exact values whenever suitable.*
☒	☐	For Bayesian analysis, information on the choice of priors and Markov chain Monte Carlo settings
☒	☐	For hierarchical and complex designs, identification of the appropriate level for tests and full reporting of outcomes
☒	☐	Estimates of effect sizes (e.g. Cohen's *d*, Pearson's *r*), indicating how they were calculated

Our web collection on statistics for biologists contains articles on many of the points above.

Software and code

Policy information about availability of computer code

Data collection	Microscopy experiments were acquired with custom Python code (https://gitlab.com/dunloplab/pycromanager/-/tree/master/pycromanager_tessie) that used the open source pycromanager Python library (v0.14) to interface with the open source MicroManager microscope control software (v2.0.1). Complete Python environment details for 284 libraries are provided for each experiment in our Zenodo archive.
Data analysis	Image analysis and features extraction was performed with our open source software DeLTA (v2.0.5 8ceb01, https://gitlab.com/dunloplab/delta). Deep learning models for timeseries prediction were implemented in Python with the open source Tensorflow library (v2.6) via its Keras API. All code to implement, train, and use the neural networks in a model predictive control framework, as well as scripts to reproduce our figures is available online (https://gitlab.com/dunloplab/deepcellcontrol)

For manuscripts utilizing custom algorithms or software that are central to the research but not yet described in published literature, software must be made available to editors and reviewers. We strongly encourage code deposition in a community repository (e.g. GitHub). See the Nature Portfolio guidelines for submitting code & software for further information.

Data

Policy information about availability of data

All manuscripts must include a data availability statement. This statement should provide the following information, where applicable:

- Accession codes, unique identifiers, or web links for publicly available datasets
- A description of any restrictions on data availability
- For clinical datasets or third party data, please ensure that the statement adheres to our policy

Datasets, processed experimental data, and trained models have been deposited on the open data archive Zenodo https://zenodo.org/record/8114649.

Research involving human participants, their data, or biological material

Policy information about studies with human participants or human data. See also policy information about sex, gender (identity/presentation), and sexual orientation and race, ethnicity and racism.

Reporting on sex and gender	Not applicable
Reporting on race, ethnicity, or other socially relevant groupings	Not applicable
Population characteristics	Not applicable
Recruitment	Not applicable
Ethics oversight	Not applicable

Note that full information on the approval of the study protocol must also be provided in the manuscript.

Field-specific reporting

Please select the one below that is the best fit for your research. If you are not sure, read the appropriate sections before making your selection.

[x] Life sciences   [ ] Behavioural & social sciences   [ ] Ecological, evolutionary & environmental sciences

For a reference copy of the document with all sections, see nature.com/documents/nr-reporting-summary-flat.pdf

Life sciences study design

All studies must disclose on these points even when the disclosure is negative.

Sample size	Sample sizes are disclosed in the figure captions and in the Main text or Methods section. All experiments were performed on thousands of single cells. For antibiotic resistance experiments we do not make statistical claims, but every gene expression level subpopulation contains at least 360 single cells per experiment, and was repeated across 3 different experiments and biological replicates to minimize the chance that results are caused by experimental variability or other factors. Sample sizes are disclosed in the figure captions and in the Main text or Methods section.<br><br>Sample sizes were not computed a priori, since we do not make any statistical claims. These sizes were dictated by the experimental throughput of our platform. For most control experiments, the sample size is 10,000 single cells. For antibiotic resistance experiments, the number of cells per controlled group and replicate ranges between 360 and 476 cells, and while again we do not make statistical claims, we provide the median response as well as the first and third quartile to illustrate distribution spread, or we provide the distribution.
Data exclusions	No data were excluded.
Replication	For antibiotic resistance experiments, all conditions were repeated 3 times on different days with different biological samples, and replicates are shown in Figure 5. Other results demonstrate the capabilities of our approach and were not replicated, although they were acquired on 10,000 cells across at least 3 separate experiments and biological samples and therefore demonstrate the robustness and reproducibility of our method.<br><br>All replication attempts were successful.
Randomization	Biological replicates were always started from overnight cultures grown from separate single colonies picked randomly from an agar plate. When different control objectives were assigned to single cells across one or multiple experiments, for example in the movie experiments or antibiotic resistance experiments, assignment was always randomized prior to starting the experiment, with a uniform distribution across cells.
Blinding	Blinding was not relevant to our study as any objective assignments were done randomly and prior to starting experiments, and no data were curated or discarded.

Because no data were curated or discarded, it is not possible for humans performing the analysis to get rid of outliers. Because all objective assignments were done randomly and prior to starting experiments, human bias in assigning cells to control objectives was not possible. For those reasons we did not need to follow a blinding protocol.

Reporting for specific materials, systems and methods

We require information from authors about some types of materials, experimental systems and methods used in many studies. Here, indicate whether each material, system or method listed is relevant to your study. If you are not sure if a list item applies to your research, read the appropriate section before selecting a response.

Materials & experimental systems

n/a	Involved in the study
☒	☐ Antibodies
☒	☐ Eukaryotic cell lines
☒	☐ Palaeontology and archaeology
☒	☐ Animals and other organisms
☒	☐ Clinical data
☒	☐ Dual use research of concern
☒	☐ Plants

Methods

n/a	Involved in the study
☒	☐ ChIP-seq
☒	☐ Flow cytometry
☒	☐ MRI-based neuroimaging
