# Supplementary material for: Deep model predictive control of gene expression in thousands of single cells
Source: Nat Commun. 2024 Mar 8;15:2148. doi: 10.1038/s41467-024-46361-1 (PMC10923782; doi:10.1038/s41467-024-46361-1)
Supplement: Supplementary file 3 — Description of Additional Supplementary Files [file 41467_2024_46361_MOESM3_ESM.pdf]

Title: Supplementary Movie 1
Description: Control strategies over time. All three cells from Fig. 3C are represented, with trajectories in the 25th percentile, median, and 75th percentile of control accuracy shown from top to bottom. Dashed gray curves show the control objective. Sliding vertical gray lines represent the current time point. Red and green background colors indicate the optogenetic stimulation sequences that were applied before the gray line, and the evolving control strategy after the gray line. Colored solid curves show measured single-cell fluorescence before the gray line, and the predicted response to the control strategy after the gray line.

Title: Supplementary Movie 2
Description: Expanding concentric sinewaves population patterning. The left panel shows the control objectives that were assigned to each cell. The center panel shows measured fluorescence values for single cells subjected to deep model predictive control in the experiment (n = 10,000 cells). The two right panels show fluorescence and optogenetic stimulations for two representative controlled cells at pixel coordinates (15, 15) and (75, 50). The movies are mirrored vertically with respect to Fig. 3D to prevent the lines indicating representative cells from obscuring the movie.

Title: Supplementary Movie 3
Description: *2001: A Space Odyssey* scene reproduction. The left panel shows the control objectives that were assigned to each cell. The right panel shows measured fluorescence values for single cells subjected to deep model predictive control in the experiment (n = 10,000 cells). Images from *2001: A Space Odyssey*, reproduced with permission from Warner Brothers, all rights reserved.

Title: Supplementary Movie 4
Description: Growth rate violin plots for all five control categories before and after tetracycline addition at t = 9h. Horizontal gray line represents the 0.3 h^{-1} "growing" vs. "dying" growth rate threshold. For visual clarity, the top and bottom 0.25% of the growth rate distributions were filtered out.
